# Psychological problems in tunisian children during the covid-19 pandemic

**DOI:** 10.1192/j.eurpsy.2022.1092

**Published:** 2022-09-01

**Authors:** A. Guermazi, F. Guermazi, A. Zouari, S. Hentati, I. Baati, J. Masmoudi

**Affiliations:** Hedi Chaker University Hospital, Sfax, Tunisia, Department Of Psychiatry A, Sfax, Tunisia

**Keywords:** psychological well-being, tunisian children, Covid-19 pandemic, psychological problems

## Abstract

**Introduction:**

The Covid-19 outbreak and the subsequent lockdown have profoundly impacted families’ daily life. Children may be among the most exposed to the psychosocial consequences of the pandemic.

**Objectives:**

To assess the psychological well-being of children during the COVID-19 pandemic.

**Methods:**

This was a descriptive study shared on social media during the period from 8 to 20 April 2021, targeting mothers of children aged 2 to 18 years. The first part included socio-demographic data of mothers and children. Then, to assess the behavior and coping skills of children and adolescents, we administered the Strengths and Difficulties Questionnaire (SDQ).

**Results:**

Our study included 65 middle-aged moms = 35.28 years. Among mothers, 1.5% reported having at least one child with a psychiatric, medical or genetic illness. The average age of the children was 8.54 years, the sex ratio was 1.03 and they were in primary school in 52.3%. Moms had talked to their child about COVID in 93.8%, using scientific data in 69.4% of cases. The total average SDQ score was 10.82; and overall mental health was at risk in 15.4% of the children. They had risky emotional symptoms in 9.2%, risky aggressive behaviors in 12.3%, risky hyperactivity-inattention symptoms in 16.9%, relationship behaviors with at-risk pairs in 24.6%, and risky prosocial behavior in 9.2% of cases.

**Conclusions:**

Researchers and government officials should be more concerned about the mental health of children who are often neglected as a result of the pandemic due to their comparatively lower mortality than older adults.

**Disclosure:**

No significant relationships.

